# An empirical comparison of the OPQoL-Brief, EQ-5D-3 L and ASCOT in a community dwelling population of older people

**DOI:** 10.1186/s12955-015-0357-7

**Published:** 2015-09-30

**Authors:** Billingsley Kaambwa, Liz Gill, Nicola McCaffrey, Emily Lancsar, Ian D. Cameron, Maria Crotty, Len Gray, Julie Ratcliffe

**Affiliations:** Flinders Health Economics Group, Repatriation General Hospital, Flinders University, A Block, 202-16 Daws Road, Daw Park, SA 5041 Adelaide Australia; John Walsh Centre for Rehabilitation Research, Kolling Institute of Medical Research, Sydney Medical School Northern, Royal North Shore Hospital, The University of Sydney, St Leonards, NSW 2065 Sydney Australia; Centre for Health Economics, Monash Business School, Monash University, Level 2, Building 75, Clayton, VIC 3800 Australia; Department of Rehabilitation and Aged Care, Repatriation General Hospital, Flinders University, C Block, 202-16 Daws Road, Daw Park, SA 5041 Adelaide Australia; Centre for Research in Geriatric Medicine & Centre for Online Health, Princess Alexandra Hospital, The University of Queensland, Level 2, Building 33, Ipswich Rd., Woolloongabba, Brisbane 4102 Australia

**Keywords:** EQ-5D-3 L, OPQoL-Brief, ASCOT, Convergent validity, Level of agreement, Community-dwelling, Older people

## Abstract

**Background:**

This study examined the relationships between a newly developed older person-specific non-preference-based quality of life (QoL) instrument (Older People’s Quality of Life brief questionnaire (OPQoL-brief)) and two generic preference-based instruments (the EQ-5D-3L Level (EQ-5D-3 L) and the Adult Social Care Outcomes Toolkit (ASCOT) in a community-dwelling population of Australian older people receiving aged care services.

**Methods:**

We formulated hypotheses about the convergent validity between the instruments (examined by Wilcoxon-Mann Whitney, Kruskal Wallis and Spearman’s correlation tests) and levels of agreement (assessed using intra class correlation (ICC) and modified Bland-Altman plots based on normalized Z EQ-5D-3 L and ASCOT utilities and OPQoL-Brief summary scores).

**Results:**

The utilities/summary scores for 87 participants (aged 65–93 years) were moderately but positively correlated. Moderate convergent validity was evident for a number of instrument dimensions with the strongest relationship (*r* = 0.57) between ‘enjoy life’ (OPQoL-Brief) and ‘social contact’ (ASCOT). The overall ICC was 0.54 and Bland-Altman scatter plots showed 3–6 % of normalized Z-scores were outside the 95 % limits of agreement suggesting moderate agreement between all three instruments (agreement highest between the OPQoL-Brief and the ASCOT).

**Conclusions:**

Our results suggest that the OPQoL-Brief, the ASCOT and the EQ-5D_3L are suitable for measuring quality of life outcomes in community-dwelling populations of older people. Given the different constructs underpinning these instruments, we recommend that choice of instrument should be guided by the context in which the instruments are being applied. Currently, the OPQoL-Brief is not suitable for use in cost-utility analyses as it is not preference-based. Given their different perspectives, we recommend that both the ASCOT and the EQ-5D are applied simultaneously to capture broader aspects of quality of life and health status within cost-utility analyses within the aged care sector. Future research directed towards the development of a new single preference-based instrument that incorporates both health status and broader aspects of quality of life within quality adjusted life year calculations for older people would be beneficial.

## Background

Australia, like many other countries, has an ageing population with the proportion of those aged 65 or over set to increase from 14 % of the total population in 2014 to 22 % by 2061 [1, 2]. This has ramifications for the levels of health and aged care services required and the ways in which these services are provided [3, 4]. In particular, growing numbers of older people are associated with a higher demand for health and aged care services which increasingly puts pressure on public funds [5]. Economic evaluation is an important technique to help decision-makers determine the relative value for money of service innovations in health and aged care [6] and is recommended for use by decision-making bodies internationally including the Pharmaceutical Benefits Advisory Committee (PBAC) and the Medical Benefits Advisory Committee (MSAC) in Australia and the National Institute for Health and Clinical Excellence (NICE) in the United Kingdom [7–9]. Previous studies have shown that for economic evaluations conducted in the aged care sector, effectiveness is best determined through the measurement of outcomes or benefits that are broad in scope [10, 11] and which older people themselves view as most valuable [12]. Maximising the quality of life of older people is also seen as a basic human right [13, 14]. Robust quality of life measurement from the perspective of older people is therefore a key requirement for economic evaluations and a highly important aspect when considering their health and aged care needs [5, 10].

A number of generic, condition-specific and older-person-specific preference and non-preference-based instruments have been used to capture quality of life in older people [5, 15]. Within economic evaluation, preference-based instruments are appealing because their application facilitates the calculation of quality adjusted life years (QALYs) which provide a common currency for assessing the benefits gained from alternative interventions in terms of both quality of life and survival [6]. While generic instruments have the advantage of being applicable to a wide range of populations and conditions [16], condition-specific (instruments focused on one particular health condition or illness) or population-specific (e.g., older person-specific) may be more sensitive and therefore more suitable for use within particular patient groups or populations [16, 17]. However, it is not always clear whether a strong relationship exists between population-specific and the more widely used generic instruments and whether the latter are as valid as the former when used in specific populations or people living with a particular condition. This study explored the convergent validity (whether scores on one instrument correlate to scores on other instruments designed to assess the same construct [18]) and levels of agreement (measuring the consistency or homogeneity of scores [19]) between a newly developed older person-specific non-preference-based instrument (Older People’s Quality of Life brief questionnaire (OPQoL-Brief) [20]) and two generic preference-based quality of life instruments (the Adult Social Care Outcomes Toolkit (ASCOT) [21] and the EQ-5D-3L Level (EQ-5D-3 L) [22]) in a population of community-dwelling older people receiving aged care services. So far, no simultaneous head-to-head comparison between all three instruments has been conducted in the literature. The results of this study will help inform decisions concerning the appropriateness of applying these instruments in various contexts within research conducted on older people.

## Methods

### Sample

Potentially eligible participants were identified by five Australian aged care provider partner organisations according to the following eligibility criteria: age ≥ 65 years, receiving community aged care services, English speaking and cognitively intact as assessed by the Global Deterioration Scale (GDS) [23] (i.e., if a GDS score ≤ 2 where 1 = normal cognition to 7 = severe dementia). Potential participants who consented to receiving further information about the study from the research team were contacted after which formal consent to participate was obtained. The study involved two main components: (i) completion of three quality of life instruments (the OPQoL-Brief, the EQ-5D-3 L and the ASCOT) and a series of socio-demographic questions, reported upon in this paper and (ii) completion of a discrete choice experiment (DCE) to elicit older people’s preferences for alternative configurations of community aged care services, the details of which are reported elsewhere [24].

The study was undertaken as a structured individual exercise completed within a group setting. Participants were asked to self-complete the three quality of life instruments independently with the research team available for the sole purpose of clarification of socio-demographic questions and/or questions within each instrument. The group setting was designed to accommodate a maximum of 20 participants with the same 3/4 researchers assisting. The groups were convened between June and December 2013 in central venues facilitated by the aged care research partners in South Australia and New South Wales [24].

### Quality of life measurement

The OPQoL-Brief is an older-person-specific measure of quality of life and is a shorter version of the original 35-item OPQoL questionnaire (OPQoL-35) [20, 25]. The construct validity of the OPQoL-35 has been demonstrated in a population of multiethnic community-dwelling older people [25, 26]. The OPQoL is a non-preference-based instrument and it was not specifically developed for application in economic evaluation. Both the OPQoL-35 and the OPQoL-Brief include health-related and broader quality of life domains [27]. The OPQoL-Brief has 13 items relating to health, social relationships, independence, control over life, home and neighbourhood, psychological and emotional wellbeing, leisure and social activities, freedom and financial circumstances. Each item has a 5-point response scale coded 1–5 from ‘strongly disagree’ to ‘strongly agree’ (with higher codes representing better quality of life). The item scores can be summed up to give summary scores ranging from 13 to 65 with higher scores indicating better quality of life [20].

The ASCOT is a generic instrument designed to capture information about an individual’s social-care-related quality of life in community and institutional settings and is applicable to individuals aged ≥ 18 years [21]. Its construct validity when used in a population of older people has been demonstrated in the literature [28, 29]. The 4 level self-completion version (SCT4) has eight domains: control over daily life, personal cleanliness and comfort, food and drink, personal safety, social participation and involvement, occupation, accommodation cleanliness and comfort and dignity [21, 30]. Each domain has four levels (‘high needs’, ‘some needs’, ‘no needs’ and ‘ideal state’) coded 1–4 with higher codes representing better quality of life. As no Australian general population-specific algorithm for the ASCOT is currently available, preference weights from the UK general population, elicited using a Best-Worst Scaling approach (a form of DCE) [21], were used to calculate a utilities ranging from −0.17 to 1 with utilities less than ‘0’ representing states that are considered to be worse than death [21, 31].

The EQ-5D-3 L is a generic health-related quality of life measure with proven construct validity when used with populations of older people [32–35]. It has five domains: mobility, self-care, usual activities, pain/discomfort and anxiety/depression. Each domain has three levels of impairment (‘no problems’, ‘some/moderate problems’ and ‘extreme’ problems) allowing the EQ-5D-3 L to distinguish between 243 states of health [36, 37]. Using the UK general population preference weights determined through the time trade off approach [36], utilities ranging from −0.59 to 1 can be attached to each of the health states with a higher utilities implying better quality of life. When the newly developed Australian general population specific scoring algorithm [38] is used, utilities ranging from −0.217 to 1 are obtained. The maximum utility of ‘1’ represents full health and a utility of ‘0’ represents dead. As with the ASCOT, utilities less than ‘0’ represent health states that are deemed to be worse than death [36, 39]. Similar to the ASCOT and for the sake of consistency, the UK-specific algorithm was applied to the EQ-5D-3 L in this study. The generic nature of the EQ-5D-3 L makes it applicable for the measurement and valuation of health related quality of life in populations of individuals aged ≥ 18 years [39, 40].

### Statistical analysis

Descriptive statistics (means, standard deviations, medians, interquartile ranges and frequencies) were generated and normality was tested using the Shapiro–Francia test [41]. The distributions of the EQ-5D-3 L and ASCOT utilities and OPQoL-Brief summary scores were skewed (Shapiro–Francia test, *p* < 0.05). Consequently, non-parametric statistical tests of differences were applied (Wilcoxon-Mann Whitney, Kruskal Wallis and Spearman’s correlation tests) [42].

The convergent validity of the EQ-5D-3 L, ASCOT and OPQoL-Brief utilities or summary scores was explored using scatter plots and an assessment of the level of association (Spearman’s correlation) between individual dimensions from each of the three instruments and between these dimensions and utilities/summary scores of comparator instruments. We also examined the distribution of mean EQ-5D-3 L, ASCOT and OPQoL-Brief utilities/summary scores across all dimension levels of comparator instruments. Correlations between 0.4 and 0.6 were considered moderate and those ≥ 0.70 strong [43]. Differences in quality of life utilities/summary scores according to demographic and other participant characteristics were tested using Wilcoxon-Mann Whitney and Kruskal Wallis tests. Characteristics examined included age in years (65–74, 75–84 and ≥ 85), gender (female versus male), living arrangements (living alone or not), highest educational attainment (below or above secondary school) and whether the participant had an informal carer or not. The ability of each instrument to discriminate between 4 levels of self-assessed general health (defined as ‘excellent or very good’, ‘good’, ‘fair’ or ‘poor’) was also examined using the Kruskal Wallis test. Based on evidence from the literature [25, 44–46], we hypothesised a priori that strong correlations would exist between dimensions that measured similar constructs e.g., between the ‘Healthy to get out/about’ (OPQoL-Brief) and the ‘mobility’ (EQ-5D-3 L) dimensions. Overall, we also expected the OPQoL-Brief and ASCOT dimension and summary scores/utilities to be more strongly correlated to each other than to those of the EQ-5D-3 L as they both measure broader aspects of quality of life while the EQ-5D-3 L is more focused upon health-related quality of life [27, 47]. As such, we also expected the EQ-5D-3 L to be more strongly correlated than the broader measures of quality of life with self-assessed general health. Further, we postulated that lower OPQoL-Brief, EQ-5D-3 L and ASCOT mean utilities/summary scores respectively would be associated with correspondingly increasing levels of severity on the dimensions of comparator instruments. Finally, we hypothesised that all three instruments would discriminate between demographic and other participant characteristics in similar directions as they all measured the broad construct of quality of life. Specific hypotheses are presented in Tables 1 and 2.

**Table 1:** Quality of life (OPQoL-Brief, EQ-5D-3 L and ASCOT) values for selected patient characteristics

Characteristics	*N* (%)	OPQoL-Brief Mean (SD)	OPQoL-Brief Median (IQR)	EQ-5D-3 L Mean (SD)	EQ-5D-3 L Median (IQR)	ASCOT Mean (SD)	ASCOT Median (IQR)
Whole sample (absolute scores)	87 (100 %)	53.931 (6.685)	53.000 (51.000–60.000)	0.515 (0.287)	0.590 (0.208–0.691)	0.852 (0.141)	0.899 (0.770–0.965)
Whole sample (Z scores)^a^	87 (100 %)	−0.000 (1.000)	−0.205 (−0.502–0.924)	−0.012 (0.990)	−0.008(−1.400–0.510)	−0.016 (0.993)	0.263 (−0.715–0.827)
Age group^b^							
65–74	15 (17 %)	52.400 (8.025)	52.000 (50.000–58.000)	0.349 (0.285)	0.487 (0.088–0.587)	0.768 (0.191)	0.776 (0.595–0.949)
75–84	41 (47 %)	53.073 (6.397)	52.000 (49.000−58.000)	0.527 (0.272)	0.620 (0.516−0.691)	0.862 (0.129)	0.905 (0.800−0.966)
≥85	31 (36 %)	55.806 (6.140)	56.000 (52.000−61.000)	0.581 (0.284)	0.590 (0.516−0.760)	0.881 (0.116)	0.908 (0.852−0.966)
*P* value			0.127		0.029		0.156
Gender^b^							
Female	57 (66 %)	53.035 (6.840)	52.000 (50.000−59.000)	0.460 (0.286)	0.587 (0.159−0.691)	0.844 (0.149)	0.890 (0.770−0.957)
Male	19 (22 %)	56.105 (5.206)	56.000 (52.000−60.000)	0.677 (0.201)	0.620 (0.587−0.796)	0.871 (0.132)	0.905 (0.829−0.975)
Missing	11 (13 %)	54.818 (7.692)	56.000 (48.000−62.000)	0.522 (0.333)	0.587 (0.193−0.760)	0.864 (0.122)	0.908 (0.769−0.957)
*P* value			0.081		0.013		0.464
Living arrangements^b^							
Living alone	51 (59 %)	54.941 (6.519)	56.000 (51.000−60.000)	0.532 (0.274)	0.620 (0.208−0.691)	0.858 (0.139)	0.904 (0.780−0.966)
Living with others	24 (28 %)	51.500 (6.311)	52.000 (48.500−56.000)	0.492 (0.299)	0.587 (0.516−0.665)	0.831 (0.159)	0.886 (0.737−0.953)
Other living arrangements	12 (14 %)	54.500 (7.416)	54.500 (49.500−61.000)	0.491 (0.335)	0.587 (0.176−0.743)	0.870 (0.118)	0.918 (0.794−0.953)
*P* value			0.028		0.445		0.426
Place of birth^b^							
Australia	49 (56 %)	53.000 (6.773)	53.000 (50.000−59.000)	0.523 (0.263)	0.620 (0.516−0.691)	0.840 (0.146)	0.877 (0.770−0.957)
UK	15 (17 %)	54.667 (5.960)	56.000 (51.000−60.000)	0.493 (0.325)	0.620 (0.159−0.691)	0.874 (0.133)	0.950 (0.800−0.966)
Other	11 (13 %)	56.455 (6.440)	56.000 (52.000−64.000)	0.538 (0.318)	0.587 (0.159−0.760)	0.861 (0.163)	0.950 (0.706−0.982)
Missing	12 (14 %)	54.500 (7.416)	54.500 (49.500−61.000)	0.491 (0.335)	0.587 (0.176−0.743)	0.870 (0.118)	0.918 (0.794−0.953)
*P* value			0.430		0.954		0.533
Education Level^b^							
Up to secondary school	44 (51 %)	53.523 (5.963)	52.000 (50.500−59.000)	0.496 (0.268)	0.587 (0.362−0.691)	0.859 (0.140)	0.902 (0.788−0.961)
Beyond secondary school	29 (33 %)	54.172 (7.691)	56.000 (51.000−59.000)	0.537 (0.303)	0.620 (0.487−0.710)	0.828 (0.154)	0.853 (0.706−0.965)
Missing	14 (16 %)	54.714 (7.021)	54.500 (51.000−60.000)	0.531 (0.325)	0.588 (0.193−0.760)	0.883 (0.114)	0.931 (0.820−0.957)
*P* value			0.406		0.328		0.398
Has Informal Carer^b^							
Yes	49 (56 %)	53.776 (7.363)	54.000 (49.000−60.000)	0.507 (0.308)	0.620 (0.159−0.691)	0.824 (0.165)	0.870 (0.706−0.966)
No	26 (30 %)	53.962 (5.024)	52.500 (51.000−58.000)	0.543 (0.225)	0.604 (0.516−0.691)	0.899 (0.080)	0.906 (0.852−0.965)
Missing	12 (14 %)	54.500 (7.416)	54.500 (49.500−61.000)	0.491 (0.335)	0.587 (0.176−0.743)	0.870 (0.118)	0.918 (0.794−0.953)
*P* value			0.802		0.898		0.154
General health^b^							
Poor	6 (7 %)	46.333 (10.40)	44.500 (42.000−52.000)	0.102 (0.258)	0.059 (−0.003−0.088)	0.711 (0.227)	0.715 (0.492−0.916)
Fair	23 (26 %)	50.565 (6.287)	51.000 (47.000−56.000)	0.361 (0.299)	0.516 (0.082−0.620)	0.804 (0.150)	0.826 (0.672−0.949)
Good	44 (51 %)	54.659 (4.927)	53.000 (51.000−58.000)	0.604 (0.184)	0.620 (0.586−0.701)	0.872 (0.118)	0.906 (0.821−0.957)
Very good or excellent	14 (16 %)	60.429 (3.589)	61.000 (59.000−62.000)	0.668 (0.286)	0.725 (0.552−0.848)	0.932 (0.084)	0.961 (0.905−0.982)
*P* value			<0.001		<0.001		0.009

*OPQoL-Brief* Older People’s Quality of Life brief questionnaire, *EQ-5D-3 L* EuroQoL EQ-5D 3 Level instrument and *ASCOT* Adult Social Care Outcomes Toolkit instrument, *IQR* Interquartile range, *SD* Standard deviation

^a^EQ-5D-3 L and ASCOT utility scores and OPQoL summary scores were first power transformed to follow a normal distribution (using the square transformation) before converting them into Z scores

^b^Based on discussions within the team and evidence from the literature, a positive relationship was hypothesised between quality of life (as measured by the OPQol-Brief, EQ-5D-3 L and ASCOT) and being younger [55, 70–73], being male [55, 73], living alone [74], having been born in Australia, a higher educational level [73, 75], having informal carer support [74] and higher self-reported general health [71]

**Table 2:** Correlation between quality of life instrument dimensions and between quality of life instrument dimensions and utilities/summary scores

	EQ-5D-3 L dimensions						ASCOT dimensions								
	Mobility	Self-care	Usual Activities	Pain/discomfort	Anxiety/depression	EQ-5D-3 L utility score	Control over daily life	Keeping clean presentable	Food and drink	How safe you feel	Social contact	Occupation/spend time	How clean and comfortable home is	How receiving help makes you feel	ASCOT utility score
OPQoL-Brief Dimensions															
Enjoy life overall	−0.24	−0.18	−0.18	−0.35	−0.18^(a)^	0.41^(b)^	0.46^(b)^	0.01	0.23^(b)^	0.12^(b)^	0.57^(b)^	0.40^(b)^	0.25	0.24	0.52^(b)^
Look forward to things	−0.17	−0.09	−0.26	−0.17	−0.23^(a)^	0.31^(b)^	0.25^(b)^	0.05	0.17	0.04	0.47^(b)^	0.29^(b)^	0.20	0.27	0.38^(b)^
Healthy to get out/about	−0.45^(a)^	−0.34	−0.43^(a)^	−0.25	−0.08	0.44^(b)^	0.21^(b)^	0.09	0.26	0.25	0.43	0.34	0.16	0.29	0.46^(b)^
Family/friends help if needed	−0.20	−0.10	−0.25	−0.26	−0.09	0.40^(b)^	0.36	0.21	0.23	0.07	0.46^(b)^	0.32^(b)^	0.09	0.33^(b)^	0.45^(b)^
Healthy enough to be independent	−0.30^(a)^	−0.34^(a)^	−0.37^(a)^	−0.21	−0.20	0.41^(b)^	0.19^(b)^	0.22	0.22	0.24	0.40	0.42^(b)^	0.12	0.05	0.40^(b)^
Can please myself in what I do	−0.25	−0.30^(a)^	−0.25	−0.13	−0.22^(a)^	0.35^(b)^	0.28^(b)^	0.15	0.06	0.08	0.29	0.27^(b)^	0.13	0.12	0.30^(b)^
Feel safe where I live	−0.19	−0.13	−0.39	−0.43	−0.03^(a)^	0.44^(b)^	0.37	0.25	0.22	0.36^(b)^	0.27	0.27	0.19	0.19	0.47^(b)^
Get pleasure from home	−0.15	−0.01	−0.15	−0.27	−0.13	0.26^(b)^	0.35	−0.04	0.13	0.01	0.36	0.22	0.26^(b)^	0.35	0.37^(b)^
Take life as it comes	−0.03	−0.15	−0.08	−0.16	−0.33^(a)^	0.25^(b)^	0.14	0.11	0.17	0.22	0.23	0.31	0.13	0.07	0.30^(b)^
Feel lucky compared to others	−0.23	−0.27	−0.12	−0.20	0.01^(a)^	0.25^(b)^	0.25	0.09	0.12	0.09	0.37	0.21	0.29	0.04	0.30^(b)^
Enough money for household bills	−0.34	−0.27	−0.23	−0.24	−0.01 ^(a)^	0.44^(b)^	0.25^(b)^	0.17	0.26^(b)^	0.17	0.21	0.15	0.24	0.33	0.38^(b)^
Have social/leisure activities I enjoy doing	−0.12	−0.19	−0.02 ^(a)^	−0.14	−0.27	0.23^(b)^	0.22	−0.10	0.18	0.04	0.36^(b)^	0.23	0.16	0.26	0.32^(b)^
Try to stay involved with things	−0.15	−0.19	−0.06^(a)^	−0.05	−0.19	0.23^(b)^	0.04	0.00	0.23	0.17	0.24	0.10^(b)^	0.16	0.01	0.21^(b)^
OPQoL-Brief summary score	−0.35^(a)^	−0.32^(a)^	−0.34^(a)^	−0.33^(a)^	−0.24^(a)^	0.53^(b)^	0.40^(b)^	0.15^(b)^	0.30^(b)^	0.22^(b)^	0.56^(b)^	0.43^(b)^	0.28^(b)^	0.30^(b)^	0.58^(b)^
EQ-5D-3 L Dimensions															
Mobility							−0.20	−0.11	−0.12	−0.19	−0.16	−0.06	−0.04	−0.09	−0.22^(a)^
Self-care							−0.06^(a)^	−0.27^(a)^	0.03	−0.35	−0.09	−0.08	−0.05^(a)^	−0.12	−0.21^(a)^
Usual activities							−0.17^(a)^	−0.23	−0.08	−0.21	−0.12^(a)^	−0.13^(a)^	0.02	−0.15	−0.23^(a)^
Pain/Discomfort							−0.33	−0.09	−0.13	−0.15	−0.22	−0.37	−0.26	−0.12	−0.37^(a)^
Anxiety/Depression							−0.08^(a)^	0.03	−0.13	−0.01 ^(a)^	−0.19	−0.10	0.04	0.00	−0.11^(a)^
EQ-5D-3 L Utility score															
							0.20^(b)^	0.17^(b)^	0.32^(b)^	0.32^(b)^	0.33^(b)^	0.26^(b)^	0.27^(b)^	0.50^(b)^	

*OPQoL-Brief* Older People’s Quality of Life brief questionnaire, *EQ-5D-3 L* EuroQoL EQ-5D 3 Level instrument, *ASCOT* Adult Social Care Outcomes Toolkit instrument

^(a)^Hypothesised negative relationships between dimension and utilities/summary scores. Note that dimension scores for the EQ-5D-3 L are ‘reverse-scored’ so that a higher (lower) score implies lower (higher) quality of life

^(b)^Hypothesised positive relationships between dimension and utilities/summary scores. Underlined correlations show all correlations ≤ |0.02|

To assess the level of agreement between the instruments, we estimated the intra-class correlation coefficients (ICC) at an individual level based on a two-way mixed-effect model where the individual effect was random and the effect of the instrument was fixed [48]. An ICC below 0.75 implies poor to moderate agreement and one above 0.75 good agreement [43]. To further study the limits of agreement between the three instruments, modified Bland-Altman plots were used. As the instruments use different rating scales leading to marked differences in the magnitude of the scores (i.e., OPQoL-Brief scores can be up to 13 times larger than those for the ASCOT and EQ-5D-3 L), Z scores of utilities/summary scores were calculated for the modified plots as recommended in the literature [49–51]. Utilities and summary scores were transformed (by squaring them) to follow a normal distribution before calculating Z scores. Of the three instruments, we hypothesised that the OPQoL-Brief and ASCOT would have the highest level of agreement given that they both incorporate broader aspects of quality of life than the EQ-5D-3 L which is more narrowly focused upon health status.

A significance level threshold of 5 % was assumed as the criterion for determining statistical significance in all analyses [52]. To account for the multiple comparisons conducted within this study, Šidák-Holm adjusted *p*-values were used for statistical tests of difference [53]. All analyses were conducted in Stata version 13.1 [48].

### Ethical approval

All procedures performed in studies involving human participants were in accordance with the ethical standards of the institutional and/or national research committee and with the 1964 Helsinki declaration and its later amendments or comparable ethical standards. Ethical approval was obtained from the Flinders University Social and Behavioural and the University of Sydney Human Research Ethics Committees.

### Consent

Informed consent was obtained from all individual participants included in the study.

## Results

### Demographic and other participant characteristics

Participant characteristics are presented in Table 1. Of the 380 potential participants initially identified as eligible for this study, 106 individuals (28 %) consented to participate and 87 individuals (82 % of those who consented to participate) provided data for this study. The mean (median) age of participants was 80 (81) years (age range was 65–93 years) and the majority (66 %) were female. The majority of participants (56 %) were born in Australia while 17 % were born in the UK; 59 % lived alone and 28 % lived with a spouse or with other family members; and 51 % had obtained a secondary school level of education or lower. The majority of participants (56 %) had an informal carer and indicated that their general health was excellent, very good or good (67 %). All study participants were receiving community support services and therefore required some assistance with activities of day to day living.

### Quality of life scores

Table 1 presents quality of life utilities (EQ-5D and ASCOT) and summary scores (OPQol-Brief) according to demographic and other participant characteristics. The mean (standard deviation) for the OPQoL-Brief scores and EQ-5D-3 L and ASCOT utilities were 53.931 (6.685), 0.515 (0.287) and 0.852 (0.141), respectively. When these results were transformed into Z scores, the mean scores for all three instruments were similar (range from −0.016 to −0.000) suggesting unsubstantial variation between the instruments. The ICC between the three instruments showed moderate level of agreement overall (0.54) (results available from authors on request). Generally, and in line with our hypothesis, the direction of the relationships between the utilities/summary scores of all three quality of life instruments and participant characteristics where statistical significance could be established was similar (Table 1). Some deviations from hypothesized directions were evident (Table 1) but these were not statistically significantly different across all three instruments. There were statistically significant differences in EQ-5D-3 L utilities (Kruskal Wallis test, *p* < 0.05) according to age group (with mean utilities increasing with age) and according to gender (mean utilities higher for males) suggesting that the EQ-5D-3 L was sensitive to age and gender differentiation. In addition, there was a statistically significant relationship between the quality of life utilities/summary scores from all three instruments and self-assessed general health indicating that all instruments discriminated well according to self-assessed general health (Kruskal Wallis test, p value < 0.01).

**Fig. 1:**
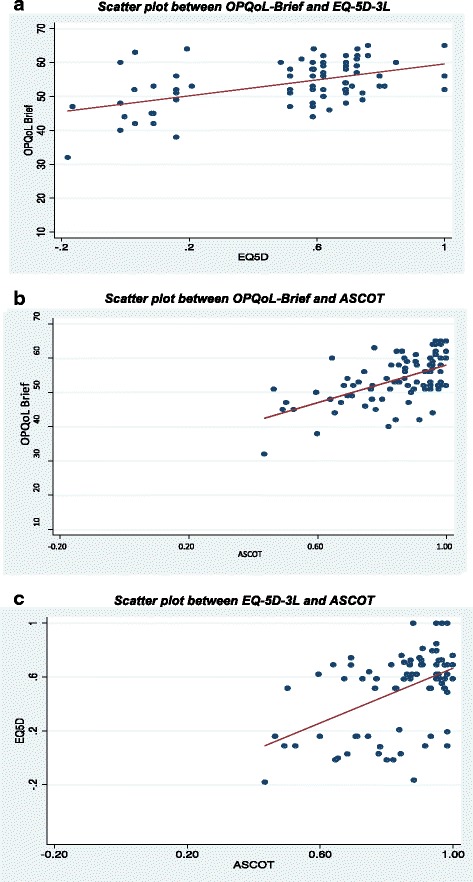
**a** Scatter plot between OPQoL-Brief and EQ-5D-3 L. **b** Scatter plot between OPQoL-Brief and ASCOT. **c** Scatter plot between EQ-5D-3 L and ASCOT

Figures 1a, b and c present scatter plots comparing utilities/summary scores between the OPQoL-Brief and EQ-5D-3 L, OPQoL-Brief and ASCOT and between the EQ-5D-3 L and ASCOT, respectively. All plots show a moderate but statistically significant positive association between the utilities/summary scores (Spearman’s correlation, p value < 0.001): *r* = 0.53 for OPQoL-Brief versus EQ-5D-3 L, *r* = 0.58 for OPQoL-Brief versus ASCOT and *r* = 0.50 for EQ-5D-3 L versus ASCOT. Our hypothesis that the highest level of agreement would be seen between the OPQoL-Brief and ASCOT was accepted. The plots also show that more individuals reported themselves to be in the best state (according to the descriptive systems of each respective instrument) for the EQ-5D-3 L (15 %) compared to the OPQoL-Brief (7 %) and the ASCOT (6 %).

The relationships between individual dimensions of all instruments and between individual dimensions of one instrument relative to utilities/summary scores for each of the comparator instruments are summarised in Table 2. The strongest evidence of convergent validity between dimensions (*r* = 0.57) was seen in the comparison between ‘enjoy life’ (OPQoL-Brief) and ‘social contact’ (ASCOT). Evidence of moderate convergent validity was seen between other similar dimensions of the OPQoL-Brief and ASCOT such as ‘enjoy life’ (OPQol-Brief) and ‘control over daily life’ (ASCOT) and between ‘healthy to be independent’ (OPQoL-Brief) and ‘occupation/spend time’ (ASCOT). In the comparison between OPQoL-Brief and EQ-5D-3 L dimensions, evidence of moderate convergent validity was also seen between the ‘healthy to get out and about’ (OPQoL-Brief) and the ‘mobility’ and ‘usual activities’ dimensions of the EQ-5D-3 L. Some apparently similar dimensions had low correlation such as ‘healthy to be independent’ (OPQoL-Brief) and ‘mobility’ (EQ-5D-3 L). Low correlation (r ≤ 0.35) was seen between all EQ-5D-3 L and ASCOT dimensions. Overall, the lowest correlations (*r* < 0.01) were seen between ‘keeping clean and presentable’ on the ASCOT and two dimensions: ‘stay involved with things’ (OPQoL-Brief) and ‘anxiety/depression’ (EQ-5D-3 L). These results show that correlations between all of the dimensions measuring similar constructs were in the direction that was hypothesised but the level of correlation was low or moderate rather than strong. Table 2 also shows that in general, higher correlations were evident between dimensions of particular instruments and utilities/summary scores of comparator instruments than those observed between individual dimensions of comparator instruments.

Tables 3, 4 and 5 present the distribution of EQ-5D-3 L, ASCOT and OPQoL-Brief utilities/summary scores across all dimension levels of comparator instruments. The majority of participants reported themselves having a good quality of life according to the classification systems of all instruments i.e., 69–93 % agreed or strongly agreed with each of the 13 statements in the OPQoL-Brief, 72–100 % reported themselves as living in the best state or had no needs on the ASCOT and 79–98 % had no or some problems on the EQ-5D-3 L dimensions. In general, and in line with our hypothesis, lower OPQoL-Brief, EQ-5D-3 L and ASCOT mean utilities/summary scores were associated with correspondingly increasing levels of severity on the dimensions of comparator instruments. Exceptions were seen for dimensions where the number of participants that chose particular responses within these dimensions was low e.g., for four OPQoL-Brief dimensions (‘I am healthy enough to have my independence’, ‘I have social/leisure activities that I enjoy doing’, ‘I try to stay involved with things’ and ‘I feel lucky compared to most people’). In terms of the ceiling effect for all instruments, the highest proportion reporting ‘strongly agree’ for the OPQoL-Brief was for the ‘I feel safe where I live’ dimension (57 %) and ranged from 22 to 53 % for the other dimensions. For the EQ-5D-3 L, 63 and 52 % reported no problems on the ‘usual activities’ and ‘anxiety/depression’ dimensions, respectively (range for other dimensions was from 15 to 23 %), while at least 70 % reported being in the ‘ideal state’ in relation to four ASCOT dimensions (‘personal cleanliness’, ‘food and drink’, ‘safety’ and ‘dignity’). The range for the percentage reporting being in the ‘ideal state’ on other dimensions was 29–63 %.

**Table 3:** Distribution of EQ-5D-3 L and ASCOT scores across dimension levels of the OPQoL-Brief

OPQoL-Briefdimensions and levels	Frequency (%)	Mean EQ-5D-3 L	Mean ASCOT
I enjoy life overall			
Strongly disagree	0 (0 %)		
Disagree	4 (5 %)	0.149	0.637
Neither agree or disagree	14 (16 %)	0.298	0.704
Agree	45 (52 %)	0.570	0.891
Strongly agree	24 (28 %)	0.601	0.903
I look forward to things			
Strongly disagree	0 (0 %)		
Disagree	2 (2 %)	−0.076	0.638
Neither agree or disagree	8 (9 %)	0.396	0.786
Agree	49 (56 %)	0.518	0.835
Strongly agree	28 (32 %)	0.588	0.917
I am health enough to get out and about			
Strongly disagree	0 (0 %)		
Disagree	15 (17 %)	0.242	0.762
Neither agree or disagree	12 (14 %)	0.500	0.738
Agree	41 (47 %)	0.560	0.883
Strongly agree	19 (22 %)	0.644	0.930
My family/friends would help me if needed			
Strongly disagree	1 (1 %)	0.159	0.599
Disagree	3 (3 %)	0.002	0.607
Neither agree or disagree	3 (3 %)	0.034	0.711
Agree	40 (46 %)	0.542	0.845
Strongly agree	40 (46 %)	0.573	0.895
I am healthy enough to have my independence			
Strongly disagree	1 (1 %)	0.639	0.747
Disagree	10 (11 %)	0.258	0.788
Neither agree or disagree	11 (13 %)	0.351	0.747
Agree	43 (49 %)	0.539	0.851
Strongly agree	22 (25 %)	0.663	0.942
I can please myself in what I do			
Strongly disagree	1 (1 %)	−0.181	0.433
Disagree	8 (9 %)	0.304	0.850
Neither agree or disagree	7 (8 %)	0.463	0.776
Agree	46 (53 %)	0.528	0.841
Strongly agree	25 (29 %)	0.603	0.913
I feel safe where I live			
Strongly disagree	0 (0 %)		
Disagree	1 (1 %)	−0.003	0.653
Neither agree or disagree	5 (6 %)	0.016	0.593
Agree	31 (36 %)	0.494	0.834
Strongly agree	50 (57 %)	0.589	0.894
I get pleasure from my home			
Strongly disagree	0 (0 %)		
Disagree	1 (1 %)	−0.181	0.433
Neither agree or disagree	5 (6 %)	0.443	0.669
Agree	35 (40 %)	0.474	0.855
Strongly agree	46 (53 %)	0.570	0.880
I take life as it comes and make the best of things			
Strongly disagree	0 (0 %)		
Disagree	4 (5 %)	0.198	0.816
Neither agree or disagree	5 (6 %)	0.361	0.762
Agree	41 (47 %)	0.530	0.816
Strongly agree	37 (43 %)	0.554	0.910
I feel lucky compared to most people			
Strongly disagree	0 (0 %)		
Disagree	1 (1 %)	0.587	0.957
Neither agree or disagree	10 (11 %)	0.387	0.765
Agree	38 (44 %)	0.460	0.820
Strongly agree	38 (44 %)	0.602	0.905
I have enough money to pay for household bills			
Strongly disagree	2 (2 %)	0.094	0.640
Disagree	1 (1 %)	−0.016	0.800
Neither agree or disagree	7 (8 %)	0.430	0.736
Agree	54 (62 %)	0.476	0.852
Strongly agree	23 (26 %)	0.694	0.910
I have social/leisure activities that I enjoy doing			
Strongly disagree	1 (1 %)	0.587	0.975
Disagree	5 (6 %)	0.241	0.679
Neither agree or disagree	11 (13 %)	0.389	0.735
Agree	42 (48 %)	0.552	0.882
Strongly agree	28 (32 %)	0.556	0.880
I try to stay involved with things			
Strongly disagree	0 (0 %)		
Disagree	2 (2 %)	0.036	0.868
Neither agree or disagree	9 (10 %)	0.354	0.722
Agree	49 (56 %)	0.553	0.862
Strongly agree	27 (31 %)	0.536	0.877

OPQoL *Brief* Older People’s Quality of Life brief questionnaire, *EQ-5D-3 L* EuroQoL EQ-5D 3 Level instrument, *ASCOT* Adult Social Care Outcomes Toolkit instrument

**Table 4:** Distribution of EQ-5D-3 L and OPQoL-Brief scores across dimension levels of the ASCOT

ASCOT dimensions and levels	Frequency (%)	Mean EQ-5D-3 L	Mean OPQoL-Brief
Control			
I have as much control over my daily life as I want	25 (29 %)	0.623	56.240
I have adequate control over my daily life	51 (59 %)	0.528	54.000
I have some control over my daily life but not enough	9 (10 %)	0.204	50.222
I have no control over my daily life	2 (2 %)	0.255	40.000
Personal cleanliness			
I feel clean and am able to present myself the way I like	69 (79 %)	0.544	54.435
I feel adequately clean and presentable	18 (21 %)	0.406	52.000
I feel less than adequately clean or presentable	0 (0 %)		
I don’t feel at all clean or presentable	0 (0 %)		
Food and drink			
I get all the food and drink I like when I want	68 (78 %)	0.545	54.941
I get adequate food and drink at OK times	15 (17 %)	0.424	51.333
I don’t always get adequate or timely food and drink	3 (3 %)	0.449	47.000
I don’t always get adequate or timely food and drink,	1 (1 %)	0.088	45.000
Safety			
I feel as safe as I want	61 (70 %)	0.568	54.754
Generally I feel adequately safe,	22 (25 %)	0.448	53.045
I feel less than adequately safe	3 (3 %)	0.079	46.667
I don’t feel at all safe	1 (1 %)	0.088	45.000
Social contact			
I have as much social contact as I want with people I like	31 (36 %)	0.565	57.387
I have adequate social contact with people	38 (44 %)	0.551	54.000
I have some social contact with people, but not enough	16 (18 %)	0.405	49.000
I have little social contact with people and feel socially isolated	2 (2 %)	−0.047	38.500
Spending time			
I’m able to spend my time as I want, doing things I value or enjoy	28 (32 %)	0.616	58.321
I’m able to do enough of the things I value or enjoy with my time	35 (40 %)	0.537	53.400
I do some of the things I value or enjoy with my time but not enough	24 (28 %)	0.366	49.583
I don’t do anything I value or enjoy with my time	0 (0 %)	0.000	0.000
Accommodation			
My home is as clean and comfortable as I want	55 (63 %)	0.588	55.782
My home is adequately clean and comfortable	29 (33 %)	0.401	50.966
My home is not quite clean or comfortable enough	3 (3 %)	0.289	48.667
My home is not at all clean or comfortable	0 (0 %)	0.000	0.000
Dignity			
The way I’m helped and treated makes me think			
And feel better about myself	64 (74 %)	0.560	55.094
The way I’m helped and treated does not affect the way I think or feel about myself	15 (17 %)	0.421	51.667
The way I’m helped and treated sometimes undermines the way I think and feel about myself	6 (7 %)	0.416	48.000
The way I’m helped and treated completely undermines the way I think and feel about myself	2 (2 %)	0.094	51.500

*OPQoL-Brief* Older People’s Quality of Life brief questionnaire, *EQ-5D-3 L* EuroQoL EQ-5D 3 Level instrument and *ASCOT* Adult Social Care Outcomes Toolkit instrument

**Table 5:** Distribution of ASCOT and OPQoL-Brief scores across dimension levels of the EQ-5D-3 L

EQ-5D-3L dimensions and levels	Frequency (%)	Mean ASCOT	Mean OPQoL-Brief
Mobility			
I have no problems in walking about	20 (23 %)	0.908	58.100
I have some problems in walking about	65 (75 %)	0.838	52.785
I am confined to bed	2 (2 %)	0.782	49.500
Self-care			
I have no problems with self-care	55 (63 %)	0.875	55.537
I have some problems washing or dressing myself	29 (33 %)	0.813	50.931
I am unable to wash or dress myself	3 (3 %)	0.801	50.667
Usual activities			
I have no problems with performing my usual activities	15 (17 %)	0.921	59.000
I have some problems with performing my usual activities	66 (76 %)	0.838	52.766
I have some problems with performing my usual activities	6 (7 %)	0.803	51.333
Pain/discomfort			
I have no pain or discomfort	13 (15 %)	0.889	55.385
I have moderate pain or discomfort	56 (64 %)	0.887	55.304
I have extreme pain or discomfort	18 (21 %)	0.724	48.611
Anxiety/depression			
I am not anxious or depressed	45 (52 %)	0.876	55.023
I am moderately anxious or depressed	40 (46 %)	0.847	53.350
I am extremely anxious or depressed	2 (2 %)	0.607	38.500

*EQ-5D-3L* EuroQoL *EQ-5D 3* Level instrument, *ASCOT* Adult Social Care Outcomes Toolkit instrument and *OPQoL-Brief* Older People’s Quality of Life brief questionnaire

**Fig. 2:**
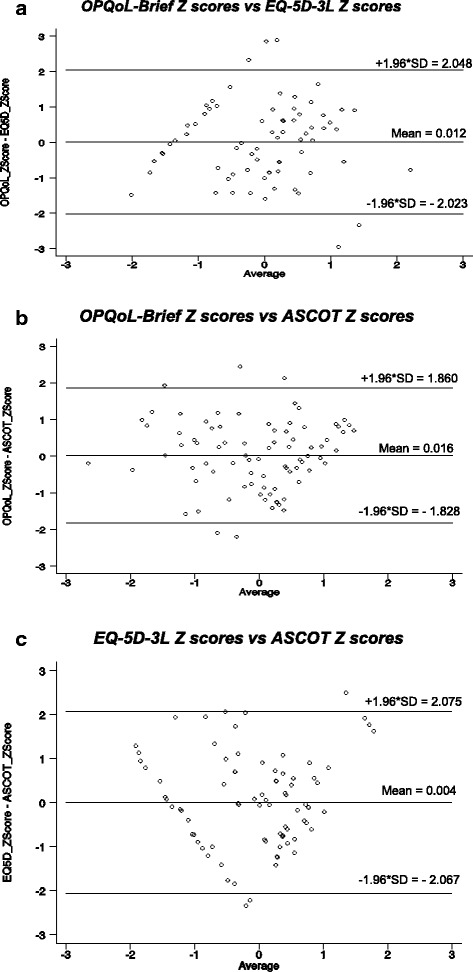
Modified Bland and Altman Plots. **a** OPQoL-Brief Z scores vs EQ-5D-3 L Z scores. **b** OPQoL-Brief Z scores vs ASCOT Z scores. **c** EQ-5D-3 L Z scores vs ASCOT Z scores

The modified Bland-Altman scatter plots in Fig. 2 show the limits of agreement between the three instruments. The plots suggest moderate agreement between all three instruments with only 3–6 % of Z scores outside the 95 % limits of agreement. As anticipated, the highest agreement (narrower limits of agreement) was between the OPQoL-Brief and the ASCOT (−1.828–1.860), then the OPQoL–Brief and the EQ-5D-3 L (−2.023–2.048) and lastly the EQ-5D-3 L and the ASCOT (−2.067–2.075) though overall the differences between the spans of the limits were marginal.

## Discussion

While the EQ-5D-3 L and ASCOT have been compared previously [28, 29, 47], this is the first study to directly compare the convergent validity and levels of agreement between the OPQoL-Brief, EQ-5D-3 L and ASCOT in a sample of community-dwelling older people receiving aged care services. As there were moderate levels of agreement between these instruments, our results support the applicability of all three instruments for measuring quality of life outcomes in populations of older people in a community setting. In general, the EQ-5D-3 L focuses more on health related quality of life while the ASCOT and the OPQoL-Brief consider broader aspects of quality of life. The findings from this study indicate that there was more agreement between the OPQoL-Brief and the ASCOT than there was between the OPQoL-Brief and EQ-5D-3 L or between the EQ-5D-3 L and the ASCOT. These findings are consistent with other studies that have shown that the ASCOT is more strongly correlated to instruments that measure broader quality of life than the EQ-5D-3 L [47].

It was found that all three instruments were able to discriminate between groups with known differences based on self-reported ratings of general health with higher mean quality of life utilities and summary scores generally reported for individuals in better general health. Unlike the ASCOT and the OPQOL-Brief, the EQ-5D-3 L was additionally able to discriminate between age groups and gender (females and males). This may suggest that age and gender are stronger predictors of health related quality of life than they are of broader quality of life. Overall, mean EQ-5D-3 L utilities increased with age in our study. This is an unexpected finding and in contrast to a number of other studies from different countries [39, 54, 55]. Further research is recommended before strong conclusions can be drawn about this relationship.

While there was statistically significant correlations in the anticipated direction between dimensions of the three instruments measuring the same constructs, this correlation was at best moderate. Further, comparisons of the utilities and summary scores also showed moderate levels of agreement overall across all instruments with the Z scores showing that the normalized mean scores were all within one standard deviation of each other. At least five reasons may help explain why the level of agreement across all instruments was not stronger. Firstly, there were differences in the ceiling effect amongst the instruments (greater for the EQ-5D-3 L compared to the ASCOT and OPQoL-Brief). Therefore, more individuals reported themselves to have been in full health on the EQ-5D-3 L than on the other two instruments. This result has also been demonstrated in other studies [37, 56] and may be due to the lower number of levels for the EQ-5D-3 L dimensions (i.e., three) compared to other instruments (five for the OPQoL-Brief and four for the ASCOT). The recent development of the new five-level version of the EQ-5D-3 L may minimize this ceiling effect [57]. Secondly, there were only small variations in responses on the dimensions of each of the instruments with most respondents (at least 85 %) classifying themselves within the top two response categories for instrument dimensions . A third explanation, linked to the developmental origins of each instrument, is that there are differences in the descriptive systems of the three instruments with the OPQoL-Brief and ASCOT having more dimensions measuring the same construct than between any other instrument comparisons. Fourthly, it is possible that other factors external to the dimensions of the instruments may have confounded some of the hypothesised relationships. For instance, it is possible that ‘mobility’ (EQ-5D-3 L) is not only dependent on being ‘healthy enough to get out/about’ (OPQoL-Brief) but may be dependent on other factors such as having the financial freedom to be mobile, feeling safe to move around or having activities to go to. Consequently and as also shown elsewhere [58], the associations between dimensions hypothesised to measure the same construct may not be as strong due to confounding relationships not being accounted for. Finally, some of the findings may also be an artefact of the relatively small sample size for this study resulting in small numbers for some of the response categories and therefore potentially exaggerated correlations [59].

Despite both low and moderate correlations being evident between individual dimensions of the three instruments, correlations between the overall utilities and summary scores were all moderate reflecting a level of correlation deemed adequate by previous studies for the purposes of determining that such instruments are interchangeable [47, 58, 60–66]. Indeed what matters to an analyst in the context of an economic evaluation is the mean value of the overall utilities/summary scores and not that of the item responses [67, 68]. We therefore conclude that given this context, all three instruments are applicable for measuring quality of life outcomes in populations of older people in a community setting.

Considering the low conceptual overlap between them, the choice of instrument may be guided by the quality of life measurement-perspective deemed to be the most appropriate in the context within which the instruments are being applied. In circumstances where quality of life needs to reflect changes in health status, the EQ-5D-3 L may be considered to be the most appropriate choice. Where an instrument is needed to measure broader quality of life (i.e., in assessing how the changes in health, aged and social care services received impact on overall quality of life), then the ASCOT should be considered if a utility-based outcome is required (i.e., in the context of cost-utility analysis) or the OPQoL-Brief if a non-preference based outcome is desired.

Study limitations include that participants in this study were essentially a self-selected group who were cognitively intact, fairly healthy and chose to participate in this research. Further, these participants represented just over a fifth of potential study participants initially identified. This therefore meant that our sample may not have been entirely representative of older people receiving consumer aged care services in Australia and that these results will need to be interpreted with this in mind. Nevertheless, we did achieve wide representation across both metropolitan and non-metropolitan areas of two Australian states and the participants exhibited a range of socio-demographic characteristics. There was also no statistically significant difference between responders and non-responders. Secondly, the comparisons made in our study focused on three instruments administered at a single time point. Further research should also be directed at longitudinal assessment to evaluate the performance of instruments in assessing change over time. Additionally, future research could consider extending the comparisons to other instruments designed for application with older people that focus on aspects of quality of life other than those captured by this study such as the ICEpop CAPability measure for Older people (ICECAP-O) [10] and the control, autonomy, self-realisation, and pleasure (CASP) measure of quality of life [69]. Finally, utility algorithms based on the UK general population were used to score the ASCOT and the EQ-5D-3 L. While an Australian general population specific scoring algorithm is available for the EQ-5D-3 L, no such algorithm is available for the ASCOT or the OPQOL-Brief. For consistency, only UK-based algorithms were used for the main analysis. However, similar findings were observed in a secondary analysis when Australian general population EQ-5D-3 L weights were used instead of the UK general population EQ-5D-3 L weights (results available from authors upon request).

In summary, we found that the OPQoL-Brief, the ASCOT and the EQ-5D_3L instruments are suitable for measuring quality of life outcomes in community-dwelling populations of older people. The results of this study support the use of both the OPQoL-Brief and ASCOT as outcome measures in economic evaluations of health and social care interventions targeted at community-dwelling older people who are cognitively intact. In this context both instruments have the attraction of considering broader aspects of quality of life beyond those incorporated within the EQ-5D-3 L which is more narrowly focused upon health status. However, because the OPQoL-Brief is currently not preference-based, its use within economic evaluation is limited.

Given their different perspectives we recommend that both the ASCOT and the EQ-5D are applied simultaneously to capture broader aspects of quality of life and health status within economic evaluations in the aged care sector. Future research to investigate the potential for the incorporation of both health status and broader aspects of quality of life within a single preference based instrument for the calculation of quality adjusted life years within economic evaluations targeted for older people would be beneficial.
